# Eukaryotic Ribosome Biogenesis: The 40S Subunit

**DOI:** 10.32607/actanaturae.11540

**Published:** 2022

**Authors:** A. A. Moraleva, A. S. Deryabin, Yu. P. Rubtsov, M. P. Rubtsova, O. A. Dontsova

**Affiliations:** Shemyakin-Ovchinnikov Institute of Bioorganic Chemistry of the Russian Academy of Sciences, Moscow, 117997 Russia; Lomonosov Moscow State University, Faculty of Chemistry, Moscow, 119991 Russia; Skolkovo Institute of Science and Technology, Moscow, 121205 Russia

**Keywords:** nucleolus, ribosome biogenesis, ribosomopathy

## Abstract

The formation of eukaryotic ribosomes is a sequential process of ribosomal precursors maturation in the nucleolus, nucleoplasm, and cytoplasm. Hundreds of ribosomal biogenesis factors ensure the accurate processing and formation of the ribosomal RNAs’ tertiary structure, and they interact with ribosomal proteins. Most of what we know about the ribosome assembly has been derived from yeast cell studies, and the mechanisms of ribosome biogenesis in eukaryotes are considered quite conservative. Although the main stages of ribosome biogenesis are similar across different groups of eukaryotes, this process in humans is much more complicated owing to the larger size of the ribosomes and pre-ribosomes and the emergence of regulatory pathways that affect their assembly and function. Many of the factors involved in the biogenesis of human ribosomes have been identified using genome-wide screening based on RNA interference. This review addresses the key aspects of yeast and human ribosome biogenesis, using the 40S subunit as an example. The mechanisms underlying these differences are still not well understood, because, unlike yeast, there are no effective methods for characterizing pre-ribosomal complexes in humans. Understanding the mechanisms of human ribosome assembly would have an incidence on a growing number of genetic diseases (ribosomopathies) caused by mutations in the genes encoding ribosomal proteins and ribosome biogenesis factors. In addition, there is evidence that ribosome assembly is regulated by oncogenic signaling pathways, and that defects in the ribosome biogenesis are linked to the activation of tumor suppressors.

## INTRODUCTION

**Fig. 1:**
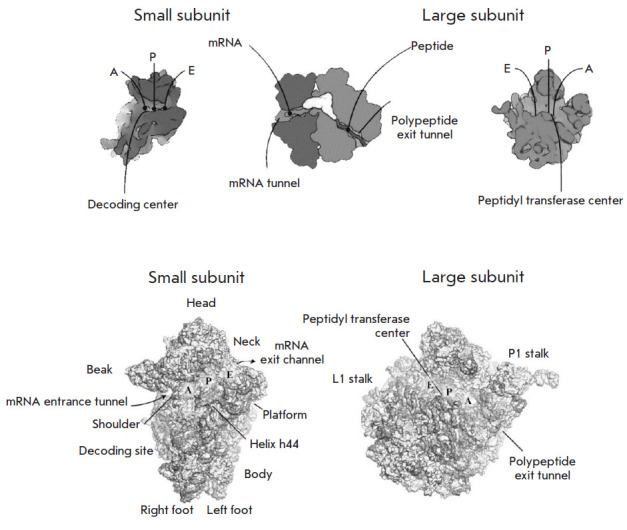
Spatial structure of eukaryotic ribosome subunits. The main functional areas of the subunits are labeled. In the small subunit, these are: (1) the channel that accommodates mRNA during translation; (2) the decoding center where codon and anticodon pairing occurs, and (3) the tRNA binding sites (sites A, P, E). Site A (aminoacyl) is occupied by the incoming aminoacyl-tRNA; site P (peptidyl) accommodates tRNA with a growing polypeptide chain (peptidyl-tRNA); site E (exit) is the place where tRNA dissociates from the ribosome. The main functional domains of the large subunit are as follows: (1) tRNA binding sites (A, P, and E); (2) the peptide exit tunnel that extends over the body of the subunit; and (3) the peptidyl transferase center (PTC). PTC is responsible for peptide bond formation and is located at the beginning of the peptide exit tunnel, in a conserved region at the interface between two subunits, which is mainly composed of rRNA. The folding of rRNA into tertiary structures and their association with ribosomal proteins generates several characteristic regions in each subunit. The main ones in the 40S subunit are the head, neck, platform, body, left foot, right foot, shoulder, and beak, as well as helix h44 of the 18S rRNA, which houses the decoding center at its base. The main tRNA binding sites (A, P, and E) are located at the interface (on the surface). The mRNA entrance tunnel is located between the head and the shoulder. The exit channel, from where the 5’-end of the mRNA egresses, is located between the head and the platform. The decoding center is located at the interface surface and includes three domains from the head, shoulder, and the h44 helix of 18S rRNA. The main features of the large subunit are the central protuberance, L1 stalk, and P stalk. The tRNA binding sites (A, P, and E) are located on the interface side, along with PTC. The latter is adjacent to the entrance to the exit tunnel, from which the nascent polypeptide chain emerges [24]

Ribosomes are molecular RNA–protein machines that ensure the translation of mRNA genetic information into proteins. Eukaryotic 80S ribosomes (S is the sedimentation constant) with a molecular mass of 4.3 MDa consist of two unequal subunits. The small subunit (40S or SSU) contains one 18S rRNA molecule and 33 ribosomal proteins (RPS or S). The large subunit (60S or LSU) comprises three rRNA molecules (25S/28S, 5.8S, and 5S) and usually 47 proteins (RPL or L) [1, 2, 3, 4]. The subunits contain several functional regions that play different roles in the translation process (Fig. 1); the sequences of mature rRNAs and the general structure of ribosomes are evolutionarily conserved. Ribosome synthesis is a fundamental process for all forms of life, and its efficiency controls the proliferative and secretory status of the cell.

During ribosome biosynthesis, the ribosomal DNA (rDNA) is transcribed, the resulting rRNA precursors (pre-rRNAs) are processed into mature molecules, which involves ribosome biogenesis factors (RBFs) and ribosomal proteins (RPs), and, finally, all components are assembled into mature ribosomes. Only an accurate sequence of all these stages leads to the formation of functional ribosomes [5]. The most complex and interesting process is the biogenesis of three rRNAs – 18S, 5.8S, and 25S/28S – which are transcribed by RNA polymerase I (Pol I) as a single, long precursor [6, 7]. The need to coordinate rRNA synthesis and processing required the formation of a specialized structure within the nucleus: the nucleolus.

## THE NUCLEOLUS IS A RIBOSOME ASSEMBLY FACTORY

Eukaryotic chromosomes usually occupy specific regions of the nucleus where genes are clustered for optimal use of the transcription machinery [8]. The synthesis of rRNA precursors and the early steps in ribosome assembly occur in a nucleus region called the nucleolus. The structural determinants of the nucleolus are nucleolar organizer regions (NORs), which are chromosomal regions where many rRNA gene repeats are grouped.

The intragenomic location of NORs depends on the species. In haploid budding yeast cells (Saccharomyces cerevisiae), the NOR occurs on chromosome 12. In humans, NORs occur on the acrocentric chromosomes 13, 14, 15, 21, and 22 [9, 10, 11]. Human rRNA gene arrays are unevenly located on the short arms of chromosomes in secondary constrictions between centromeres and telomeres [12, 13]. During eukaryotic division, nucleoli assemble in the end of mitosis and remain functionally active throughout the entire interphase, disintegrating at the beginning of the next mitosis. Ribosome production alters during the cell cycle, reaching a maximum in the G2 phase [14]. Nucleolar morphology significantly depends on the growth conditions and physiological status of the cell [15]. The nucleolar size correlates with the proliferative activity of the cell; nucleoli in rapidly dividing cells are larger than those in slowly dividing cells [16]. The nucleolar volume in most tumor cells is enlarged compared to that in their progenitors [17].

**Fig. 2:**
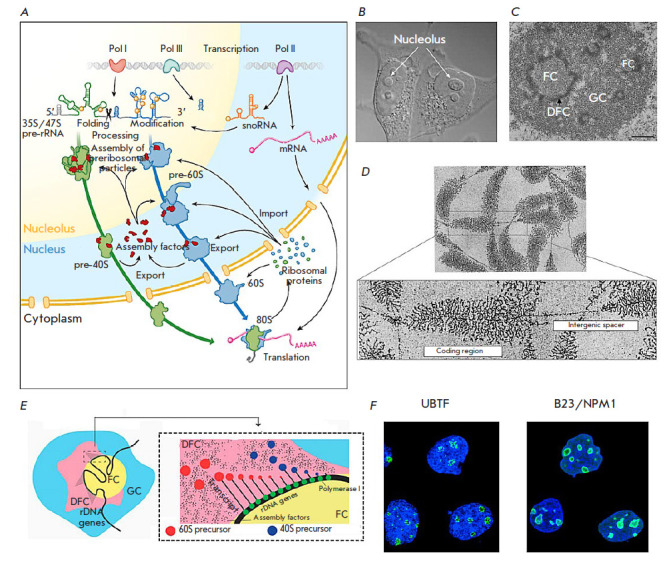
Eukaryotic ribosome biogenesis. (*A*) General scheme [5]; (*B*) Nucleoli of HeLa cells, phase contrast [18]; (*C*) Electron micrograph of the HeLa cell nucleolus: granular component (GC), fibrillar center (FC), and dense fibrillar component (DFC) [19]; (*D*) Tandem repeats of ribosomal genes and transcribed rRNA of the newt oocyte were stained using the Miller method. (http://www.cellimagelibrary.org); (*E*) Mutual arrangement of subdivisions of human nucleoli [13]; (*F*) Localization of the ribosome processing factors UBTF in DFC and B23 in GC of the nucleoli of human A-43 cells stained with specific antibodies (https://www.proteinatlas.org/)

The nucleolus is the largest part of the nucleus, which is not separated by a membrane from the nucleoplasm; its volume accounts for 20–25% of the nucleus in higher eukaryotes. According to electron microscopy (EM), finer structures in the nucleolus correspond to the main stages of ribosome biogenesis. The fibrillar center (FC), a dense fibrillar component (DFC), and the granular component (GC) can be distinguished (Fig. 2).

Ribosome biogenesis is a vector process that begins with rRNA synthesis at the interface between FC and DFC, continues in DFC, and ends in GC. Thus, FCs contain rDNA, Pol I and DNA topoisomerase I subunits, and the upstream binding factor [18]. In DFC, synthesis and early stages of rRNA processing occur. For example, fibrillarin, Nopp140, and small nucleolar RNAs (snoRNAs) are involved in the early stages of rRNA processing and are localized in DFC [18, 19, 20, 21]. Mutation in the main casein kinase 2 (CK2), a key protein of the granular component of human nucleophosmin (NPM/B23)) phosphorylation site leads to the detachment of GC from DC/DFC, which indicates a transition between the stages of pre-40S and pre-60S ribosome subunit assembly at the border between DFC and GC. The nucleolar stage of SSU and LSU precursor assembly in yeast, which continues with export to the nucleoplasm, takes a different amount of time. For example, SSUs leave the nucleolus approximately 10 min after the start of assembly, almost twice faster than LSUs [21, 22, 23]. The distribution of ribosome maturation stages over different structures of the nucleolus architecture in higher eukaryotes remains poorly understood.

Recently, new mechanisms that underly the nucleolus formation control have been proposed. They are based on the multiphase organization related to liquid–liquid phase separation [13]. Pre-rRNAs are supposed to recruit certain proteins, which leads to phase separation. The spatial separation and physical and compositional features of subnucleolar phases can optimize pre-rRNA processing, providing targeted transport and hierarchy of pre-ribosome assembly processes. Early stages of pre-rRNA processing and covalent modification of highly conserved rRNA residues (ribose and base methylation and pseudouridylation), which are essential for the structural organization of ribosomes and regulation of the translation process [24, 25, 26], occur in DFC (Fig. 2). The external GC acts as a temporary "quarantine" for misfolded nuclear proteins that accumulate under stressful conditions [13, 27].

Homologues of ~90% of yeast nucleolar proteins have been identified in the human nucleolus proteome [28]. According to the classification of nucleolar proteins functions, ~30% of them are associated with ribosome biogenesis [29]. Dysregulation of nucleolar proteins may lead to cell cycle arrest and apoptosis or, conversely, promote cell transformation and accelerate proliferation [30]. RPs also play an important role in the assembly process, as they are believed to stabilize the secondary rRNA structure, promoting the formation of cleavage-competent tertiary structures, and prevent misfolding. RPs from HeLa cells (32 proteins) may be classified into two categories depending on their involvement in the early or late stages of processing. The moment of RP attachment to pre-ribosomes correlates with their contribution at the stage of RNA precursor cleavage [6]. Pre-rRNA processing is a determining factor in the formation of mature functional ribosomes, and, in this review, we will focus on sequential maturation of the Pol I transcription product, a common precursor of 18S, 5.8S, and 25S/28S rRNAs.

## RIBOSOME BIOGENESIS

**Main processing stages and differences in the structure of yeast and human rRNA precursors**

**Fig. 3:**
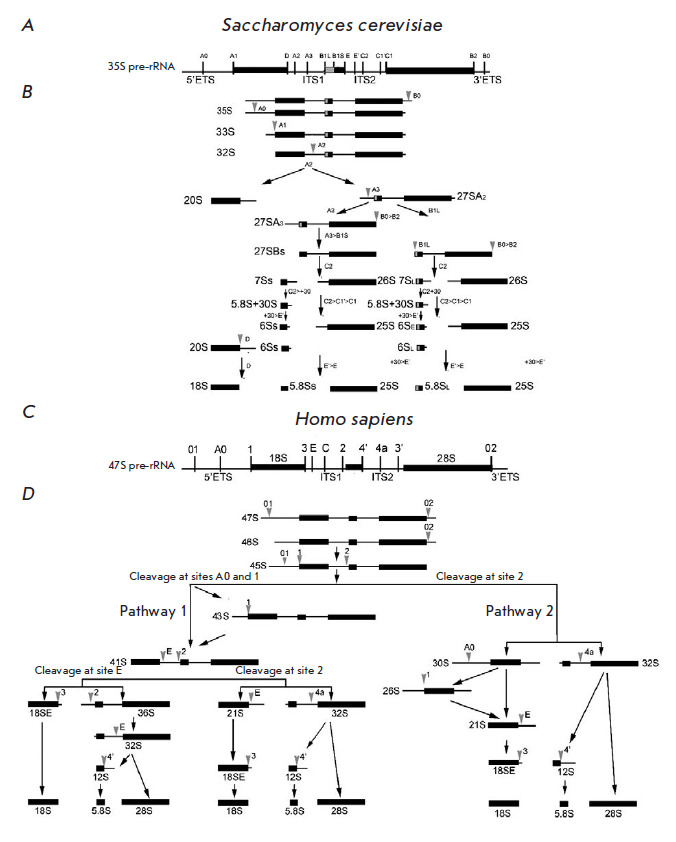
Maturation pathways of the yeast 35S pre-rRNA transcript (*A*) and human 47S pre-RNA transcript (*C*). Three of the four rRNAs (18S, 5.8S, and 25S (in yeast)/28S (in humans)) are synthesized by Pol I as a single long transcript. The coding sequences of mature rRNAs are flanked by 5’- and 3’- ETS, ITS1, and ITS2 non-coding spacers. The schematic shows the relative position of known and predicted cleavage sites. (*B*) Processing of pre-rRNA in budding yeast. (*D*) A simplified schematic of human pre-rRNA processing. A primary transcript, 47S pre-rRNA, is initially cleaved at both ends at sites 01 and 02 to form the 45S precursor that is processed via two alternative pathways [6]. ">" (e.g., C2>C1’>C1) denotes sequential shortening of the appropriate 3’- or 5’-ends of the pre-rRNA by nucleases

Transcription of rRNA genes leads to the formation of a pre-rRNA precursor (35S in yeast and 47S in human cells), which includes 18S, 5.8S, and 25S/28S rRNA sequences flanked with external transcribed spacers (5’-ETS and 3’-ETS) and separated by internal transcribed spacers (ITS1, between 18S and 5.8S; ITS2, between 5.8S and 25S/28S) (Fig. 3). During sequential maturation of pre-rRNAs, RNA intermediates are formed. Folding of long rRNAs is a difficult task, because their size allows these molecules to be in alternative stable non-functional structures. Unlike relatively weak interactions that maintain the spatial structure of proteins (e.g., alpha-helices and beta-sheets), approximately half of the folded rRNA structure is composed of the more stable A-form double helices [13]. Therefore, the existence of extended non-transcribed ETS and ITS spacers (about half of the primary rRNA transcript), which only complicate the structure of rRNA precursors, seems illogical. The role of external spacers is probably to reduce the risk of rRNA mutations owing to RNA polymerase errors, which more often occur in the 5’- and 3’-termini of transcripts. Although spacer sequences differ, their ends are evolutionarily conserved and fold into several hairpin structures [31]. The sequences of the noncoding spacer ITS1 are less conserved [32], which complicates any prediction of cleavage sites even in closely related species. Mammalian ITS1 sequences are usually 2–3 times lengthier and possess a much higher G + C content than yeast ones (mice, 70.1%; yeast, 35.2%) [33, 34].

Because rRNA performs both structural and catalytic functions, it is not surprising that the key aspects of ribosomal subunit maturation include the formation of structural domains in rRNA, folding into the three-dimensional structure, and concomitant excision and removal of spacers from compound RNP complexes. In addition, the large subunit precursor pre-60S should include the 5S rRNA and its associated ribosomal proteins (Fig. 3) [6]. The RNA–protein composition of ribosomal precursor complexes is studied using a combination of biochemical approaches; in particular, Northern blotting, rapid amplification of cDNA ends (RACE) combined with DNA sequencing, Western blotting with antibodies to RPs and RAFs (ribosome assembly factor), as well as mass spectrometry and high-resolution cryo-electron microscopy (cryo-EM) to characterize secondary- and tertiary-structure elements. Combination of these methods enables mapping of the main pre-rRNA cleavage sites in yeast, mice, and humans [6, 35] and the elucidation of the protein–nucleic acid composition and 3D structure of individual complexes.

**Saccharomyces cerevisiae ribosome biogenesis, rRNA processing**

Figure 3 A, B provides a schematic for cleavage and truncation of the ends of S. cerevisiae pre-rRNA. The RNase III homologue Rnt1 co-transcriptionally hydrolyzes 3’-ETS at the B0 site in primary 35S pre-rRNA transcripts [35, 36, 37, 38]. Subsequent cleavage at the A0, A1, and A2 sites is interdependent (Fig. 3B), and in fast growing cells, co-transcriptional cleavage at ITS1 occurs in 50–70% of cases. Cleavage at A0, A1, and A2 is performed by the SSU processome containing snoRNA U3. The endonucleases Utp24 and Rcl1 hydrolyze pre-rRNAs at the A1 and A2 sites, respectively [39, 40]. The products 20S and 27SA2 further form SSU and LSU, respectively. 20S enters the cytoplasm, turning into 18S after cleavage at the D site by Nob1 nuclease (Fig. 3).

Maturation of the 27SA2 pre-rRNA leads to the formation of alternative 27SB forms which differ by additional 7–8 nucleotides at the 5’-end. The RNase MRP cleaves approximately 80% of 27SA2 at the A3 site, and Rat1–Rai1 (Rrp17) proteins truncate 27SA2 to the B1S site (probably, together with 5’–3’-exonuclease Xrn1). The remaining 20% of 27SA2 is cleaved by an unknown RNase at the B1L site, with hydrolysis at B1L and B2 occurring simultaneously (Fig. 3). Cleavage of 27S B1S and B1L at the C2 site within ITS2 results in the formation of 7S pre-rRNA (5.8S precursor) and 26S pre-rRNA (25S precursor). The RNA exosome, which comprises the Rrp6 and Ngl2 subunits and Rex exonuclease, truncates the 7S pre-rRNA to the E site which corresponds to the 3’- end of 5.8S. The 3’-end of 5.8S rRNA is finally formed in the cytoplasm, probably with involvement of Ngl2 that acts as a nuclease both in the nucleus and in the cytoplasm. Impairment of pre-rRNA processing kinetics at sites between A0 and A2 leads to aberrant rRNAs, which occurs upon knockdown of the genes of proteins essential for the processing of the 27SA2 pre-rRNA at the A3 site: Cic1, Erb1, Nop7, Nop12, and Nop1 (Fig. 3) [41]. Non-optimal growth conditions and mutations interfering with SSU or LSU synthesis affect the order of RNA cleavage [42], which leads to accumulation and cleavage of the 35S pre-rRNA immediately at the A3 site, but not at A0, A1, and A2, to form 23S, an aberrant product inappropriate for 18S rRNA maturation [43].

Processing of pre-rRNA and attachment of ribosomal proteins require many auxiliary RAFs, in particular RNA helicases, ribonucleases, GTPases, ATPases, RNA chaperones, and non-enzymatic proteins [44]. Some RAFs temporarily block transitions between the structures of subparticle precursors, preventing rRNA misfolding or premature binding of RAFs and RPs, which are required at later stages of assembly. As subunits mature structurally, RAF binding mimics the binding of translation factors or substrates (e.g., tRNA or mRNA) and prevents involvement of immature particles in translation initiation.

The earliest, large RNP–90S complex is formed co-transcriptionally. The structures of early intermediates were visualized using cryo-EM methods in [45, 46]. Simultaneously with transcription, rRNA undergoes covalent modifications, most of which occur in functionally important domains and are also believed to be essential for the rRNA structure [47]. In the three-dimensional structure of the human 80S ribosome, 130 rRNA modifications (methylation and pseudouridinylation) were revealed by cryo-EM [48]. Pseudouridinylation is performed by Cbf5, Gar1, Nop10, and Nhp2 synthases belonging to the H/ACA snoRNP class, while methylation of 2’-O-ribose is performed by C/D-box snoRNA proteins, such as Nop1 methyltransferase (fibrillarin in humans), Nop56– Nop58 heterodimer, and Snu13 [49, 50]. Probably, modifications occur during transcription and initial folding of pre-rRNAs because snoRNAs hybridize more efficiently to partially unfolded pre-rRNA. Some snoRNAs required for ribosome assembly do not modify pre-rRNAs but stabilize structures that benefit the assembly and maturation of pre-ribosomal particles. Subunit precursors are also modified by specific snoRNA-independent methyltransferases [5, 51] and acetylases [52].

The assembly of yeast ribosomes involves 19 RNA helicases, including DEAD-box and DEAH-box helicases, but their role in this process remains unclear [53]. Three helicases (Has1, Mtr4, and Prp43) are involved in the assembly of both subunits [54, 55]. The energy in this process is provided by GTPases (Bms1, Nog1, Nog2, Nug1, Lsg1, and Efl1), ATPases (Rio1, Rio2, and Fap7), and AAA ATPases (Mdn1, Drg1, and Rix7) [56]. The role of these factors is to maintain the irreversibility of the assembly processes.

**Yeast ITS2 processing**

**Fig. 4:**
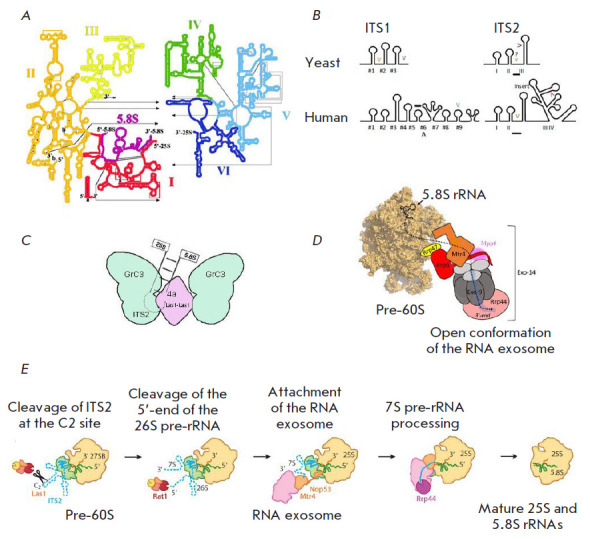
Structure and maturation of yeast pre-rRNA. (*A*) The 25S rRNA contains six domains (I–VI). The 5.8S rRNA (shown in black) forms complementary interactions with domain I of the 25S rRNA (adopted from https://crw-site. chemistry.gatech.edu/). (*B*) Secondary structures of yeast and human ITS1 and 2. Cleavage sites are denoted by "V." Predicted sites are marked by "?"; the human exonuclease binding sites are underscored. (*C*) Model of ITS2 processing by RNase PNK [49, 52]. (*D*) Interaction of the nuclear RNA exosome with pre-60S [78]. (*E*) Removal of ITS2 from the pre-60S particle by RNA processing enzymes. Intermediates during ITS2 removal are shown [5]

ITS2 is a structural element that serves as the basis for several stages of 60S assembly, similar to 5’-ETS in the early stages of 18S rRNA maturation. Removal of ITS2 located between 5.8S and 25S rRNAs is considered one of the most difficult steps in ribosome assembly. Despite its short length (only a few hundred nucleotides), yeast ITS2 is highly structured and forms a dense and conserved core [57, 58]. An in vivo study of the pre-rRNA structure showed that ITS2 folds into a long hairpin structure with the C2 cleavage site at the stem end (Fig. 4) [59]. Disturbances of the hairpin sequence and structure block ITS2 processing, indicating its key importance in ribosome assembly [60, 61]. According to the cryo-EM structure, the pre-60S ITS2 base structure forms paws and involves several assembly factors [62, 63, 64]. There is a model where ITS2 rRNA and associated biogenesis factors (Nsa3, Nop7, Erb1, Rlp7, Nop15) facilitate hybridization of the 25S rRNA domain I and 5.8S. This model is supported by data indicating that mutations in these proteins inhibit ITS2 processing at early stages [65, 66, 67, 68].

There are three phases of ITS2 processing: (1) cleavage and phosphorylation of the C2 site by the Las1–Grc3 complex, (2) hydrolysis of the 5’-end by Rat1 exonuclease, and (3) hydrolysis of the 3’-end by the RNA exosome (Fig. 4). Processing of ITS2 activates a tetrameric enzymatic complex consisting of two HEPN Las1 endonuclease and Grc3 polynucleotide kinase dimers (they function only as dimers; the level of the proteins is co-regulated) [69]. The N-terminal HEPN domain comprises the RφxxxH catalytic motif (φ is H, D, or N, and x is any amino acid) [70]. Depletion of mammalian LAS1L (Las1-like), an ortholog of yeast Las1, leads to inhibition of ITS2 processing and cell proliferation [71]. Depletion of yeast cells in Las1 also blocks ITS2 processing, which indicates conserved functions of Las1 in ITS2 processing in eukaryotes [69, 72]. C2 cleavage and phosphorylation are related processes; phosphorylation prevents re-ligation of C2 cleavage products: 7S pre-rRNA with 2’-3’-cyclophosphate and 26S pre-rRNA with 5’-hydroxyl [60, 61, 73]. Grc3 recruits the 5’ → 3’ exonuclease Rat1 (mammalian Xrn2) to the C2 site of the 26S pre-rRNA [61, 74, 75]. Rat1/Xrn2 (non-sequence-specific) hydrolyzes a single-stranded RNA with a terminal 5’-monophosphate in the 5’ → 3’ direction [76]. Yeast Rat1 and its activating cofactor, nuclease Rai1, form a dimeric complex that binds Las1–Grc3 via Grc3 [73] in pre-60S particles [73, 76, 77]. Binding between Rat1-Rai1 and Grc3 is rather weak, which implies additional interactions at the C2 site [60, 73, 78]. The amino acid sequences of Grc3/Nol9 and Rat1/ Xrn2 are very conserved, suggesting conservation of Grc3-dependent recruitment of Rat1 to the C2 site. Details on a molecular interaction between Grc3/Nol9 and Rat1/Xrn2 are unknown, which complicates our understanding of the mechanism of ITS2 5’-end truncation.

The RNA exosome hydrolyzes the 3’-end of the 7S pre-rRNA after cleavage of the ITS2 5’-end (Fig. 4). The RNA exosome is a multisubunit 3’ → 5’ ribonuclease complex that hydrolyzes any known forms of RNA [79, 80]. It comprises a core of 9 subunits (Exo-9) which form a two-layer ring with a central channel (Fig. 4) [78, 79, 81, 82, 83]. The Exo-9 core lacks catalytic activity and requires multiple partners to degrade RNA. The catalytic activity of the RNA exosome depends on the Rrp44 enzyme possessing the endonuclease and 3’ → 5’ exonuclease activities [84, 85]. Rrp44 binds the Exo-9 core to form the Exo-10 complex [79, 81] that interacts with additional 3’ → 5’ nuclease, Rrp6, to form Exo-11 [82, 86, 87, 88, 89]. Additional proteins – Mpp6, Rrp47, and Rrp6 – recruit the Mtr4 cofactor, enhancing binding of the complex to pre-ribosomes, into the exosome. The interaction between Mtr4 and Nop53 or Utp18 directs Exo-11 to ITS2 and 5’-ETS, respectively (Fig. 4E) [90]. The helicase Mtr4 unwinds the ITS2 end in the 3’ → 5’ direction [91, 92, 93], enabling Rrp44 to hydrolyze the 3’-end of the 7S pre-rRNA. The resulting transcript encodes 5.8S with an additional 30 ITS2 nucleotide tag (Fig. 4) [92, 94, 95]. Further, Rrp6 nuclease cleaves ITS2 to form the 6S pre-rRNA [92]. A recent cryo-EM structure of the RNA–exosome revealed that it undergoes structural rearrangements upon binding to pre-60S [78, 96], forming a channel inside the RNA–exosome core, through which the 7S pre-rRNA reaches the Rrp44 exonuclease active site [78, 95, 96] (Fig. 4).

**Human rRNA processing**

Processing of human 18S rRNA includes more steps than those in yeast cells [23, 35] (Fig. 3). At the first stage of processing, the primary 47S transcript (Fig. 3) is truncated at both ends at the A0 (or 01) and 02 sites, which leads to the release of 5’- and 3’- ETS, respectively, and the formation of a 45S pre-rRNA precursor (Fig. 3) that is then truncated via two alternative pathways. In human cells, cleavage of the 47S pre-rRNA at the A0 and 02 sites is coordinated in time. Perturbation of this coordination leads to the accumulation of a 46S intermediate. The 45S pre-rRNA is processed via parallel pathways (1 and 2) to form numerous intermediates (Fig. 3D). Also, an important role in the processing (along with endonucleases) is played by exonucleases which truncate rRNA at the ends.

Some human pre-rRNA molecules are probably cleaved co-transcriptionally, as in yeast cells. In mammals, pre-rRNAs are supposed to be co-transcriptionally cleaved only at the A’ site [97]. It is worth noting that there are conditions that favor one of the alternative pathways. For example, mutations in U3 or U8 snoRNAs disrupt the order of pre-rRNA cleavage [98]. The first 47S pre-rRNA cleavage occurs at site 01, located several hundred nucleotides downstream of the transcription start, at the 5’-ETS binding site for C/D snoRNA U3. The order of precursor cleavage also depends on the species and type of cells, physiological conditions, and cell cycle stages and is disturbed in disease [6, 99, 100, 101].

The key RAFs and RPs involved in pre-rRNA processing and an analysis of the differences in the yeast and human rRNA processing machineries will be addressed when considering the assembly of certain SSU and LSU precursors.

**Fig. 5:**
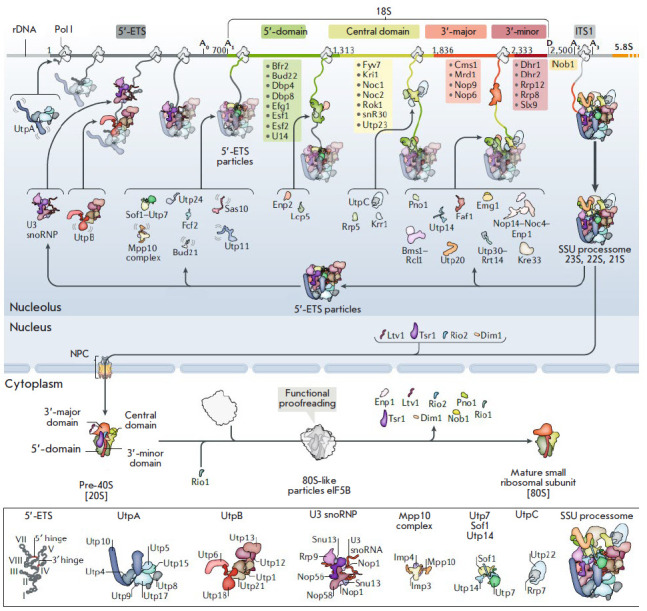
The factors and complexes involved in the assembly of the yeast small subunit. The main stages of 40S subunit maturation in yeast are shown. (Top) rDNA with the main domains of the 18S rRNA: 5’-ETS, ITS1, 5’-central, 3’-major, and 3’-minor domains. Also, sites (A0, A1, D, and A2) are shown. (Below) Intermediate pre-ribosomal particles: 5’-ETS complex, SSU processome, and pre-40S. The intermediate components of pre-rRNA complexes are shown in square brackets under each particle. Assembly factors and complexes for which (not transparent) structures have been identified are depicted as cartoons, whereas those for which no structures are known are indicated only with text. Proteins that joined the growing SSU processome at an earlier stage are shown as transparent to highlight new components (not transparent). Adopted from [44]

Although rRNA synthesis and maturation are the key events in the ribosome subunit biogenesis, there are other important aspects to this process: e.g., attachment of ribosomal proteins and RAFs at certain stages (Fig. 5). The ribosome assembly is based on four main principles: (1) a gradual decrease in the conformational freedom of pre-rRNA; (2) the sequence and temporal dynamics of binding of individual assembly factors provided by molecular mimicry and molecular switches; (3) the irreversibility of key checkpoints, which depends on energy consumption and enzymes that change the RNA length and structure; and (4) structural and functional correction of the active sites of both ribosomal subunits.

**Assembly of 90S pre-rRNP**

**Fig. 6:**
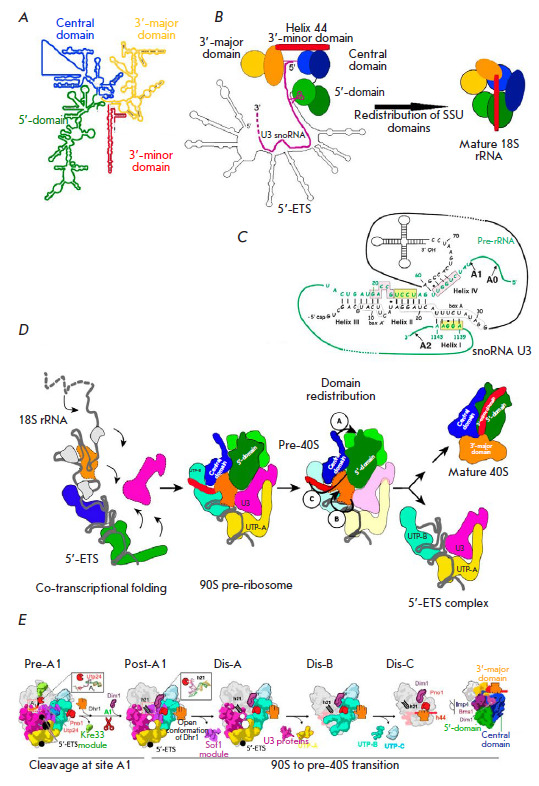
Domain rearrangements during maturation of the 40S subunit. (*A*) The 18S rRNA contains the following domains: 5’-domain, central domain, 3’-major domain, and 3’-minor domain (adopted from https://crw-site.chemistry. gatech.edu/). (*B*) Schematic of the SSU processome (left) and mature 18S (right). 18S domains are shown in different colors: 5’-domain (green), central domain (blue), 3’-major domain (yellow), 3’-minor domain (red rectangle), and U3 RNA (pink line) [13]. (*C*) Base-pair interactions between the U3 snoRNA and the 18S region of the pre-rRNA in yeast. Three interactions between Box A and Box A’ in the U3 snoRNA and three 18S regions of the pre-rRNA, which are involved in the formation of the central pseudoknot structure in the mature 18S rRNA [23, 35]. (*D*) Model of 90S formation and its transformation into pre-40S. The snoRNP modules UTP-A (yellow), UTP-B (blue), and U3 (pink) bind co-transcriptionally to the 35S pre-rRNA. Further compaction leads to 90S complex formation. General folding of the 5’-domain of the 18S rRNA resembles the mature conformation, but transformation of the pre-40S preribosome 90S into the mature 40S subunit requires structural rearrangements in the central, 3’-major (orange), and 3’-minor (red) domains [23, 35]. (*E*) Schematic of 90S transformation into pre-40S upon cleavage at A1. Assembly factors and selected proteins are colored and labeled accordingly. The helicase Dhr1 is shown as a grasping hand representing open and closed conformations.

As the transcript is released from contact with Pol I, the 5’-ETS rRNA folds into stem–loop structures, providing a platform for the attachment of RAFs and RPs and for the folding of four SSU domains (Fig. 6A). Because these structures are formed co-transcriptionally, they provide binding sites for a number of RAF complexes, in particular the molecular chaperones UTP-A, UTP-B, and U3 snoRNA, ordering the assembly. At this stage, the hairpin structures formed by 5’-ETS play the main role (Fig. 6 A, B) [44]. A significant variability in the primary structures of 5’-ETS and ITS in different species indicates the key role played by the spatial structure formed by these elements in ribosome biogenesis [102]. By pairing with rRNA bases, snoRNA U3 renders the rRNA structure rigid. In the 90S cryo-EM structure, a partially prominent complex of the 3’-terminal part of the U3 snoRNA with the main C/D-box factors (Nop1, Nop56, Nop58, Snu13, Rrp9) is observed. The single-stranded 5’-end of U3 penetrates deep into the SSU particle, hybridizing with the short, conserved nucleotide sequences of 18S rRNA and 5’-ETS (Fig. 6B). This process is accompanied by the formation of 5’- and 3’-loops and promotes excision of the 18S pre-rRNA owing to the formation of Box A and Box A’ [44, 103, 104, 105, 106, 107, 108, 109] (Fig. 6B). The close proximity of these sites to the 5’ region of snoRNA U3 provides a crucial spatial constraint that dictates the topology of the maturing particle. The complex comprising the folded 5’-ETS 18S pre-rRNA with an uncleaved A1 site and early RPs is incorporated into the structure formed by biogenesis factors (~60 proteins) and snoRNA U3 (Fig. 6, Table). Timely cleavage at the A1 and A2 sites requires U3-dependent formation of the 35S pre-rRNA conformation that prevents the formation of the central pseudoknot, a characteristic structure located at the decoding center in mature 18S rRNA (Fig. 6). A number of early RAFs (Utp11, Sas10, Mpp10, and Fcf2) (Fig. 5) limit the pre-rRNA domains inside the particle by binding either to the protein or to RNA elements. In the 90S pre-ribosome, only the 5’-domain has a conformation close to that of the mature one and, accordingly, contains RPs (Fig. 6). The central domain is only partially visible, and the 3’-terminal domains cannot be distinguished in the 90S structure. Thus, folding of the nascent 18S rRNA occurs in the direction from the 5’-end to the 3’-end but is blocked at intermediate stages involving additional RAFs (Fig. 5, Fig. 6). The 90S subparticle comprises the GTPase Bms1. After hydrolysis of GTP, this enzyme is believed to initiate the conformational changes necessary for pre-rRNA processing and transformation of 90S into the pre-40S subunit. According to this hypothesis, Bms1 is located at the interface of several pre-18S domains and comes into contact with several RAFs that stabilize the 90S intermediate (Fig. 5).

**Table:** Small ribosomal subunit assembly factors [44]

Ribosome biogenesis factors of the SSU component in Saccharomyces cerevisiae					
Cluster number			Human S.	cerevisiae	Function
2	2	8	DDX47	Rrp3	DEAD-box-helicase
6	2	2	DDX49	Dbp8	DEAD-box-helicase
1	1	1	DDX42	Rok1	DEAD-box-helicase
1	1	1	EIF4A3	Fal1	DEAD-box-helicase
2			Rrp36	Rrp36	Structural
11	11		MYBBP1A	Pol5	Same
2	2		ABT1	Esf2	«
1	1	1	Esf1	Esf1	«
3			Utp23	Utp23	«
4	4	11	NOC2L	Noc2	«
8	3	3	RBM19	Mrd1	«
		2	C14orf21	Nop9	«
1			Rrp8	Rrp8	rRNA methyltransferase
				H/ACA components	
		2	Gar1	Gar1	Pseudouridine synthase cofactor
2	2		Nhp2	Nhp2	Pseudouridine synthase cofactor
			Nop10	Nop10	Pseudouridine synthase cofactor
				UtpA complex	
2	2	2	CIRH1A	Utp4	Structural
2	2	5	WDR43	Utp5	Same
2	2		HEATR1	Utp10	«
1	1	1	Utp15	Utp15	«
5	5	2	WDR75	Utp17/Nan1	«
				UtpB complex	
2	2	2	PWP2	Utp1/Pwp2	«
2	8	8	Utp6	Utp6	«
2	2	2	WDR3	Utp12	«
2	2	2	TBL3	Utp13	«
2	2		Utp18	Utp18	Structural, has the exosome binding motif
2	2	2	WDR36	Utp21	Structural
				U3 snoRNP	
2	2	2	Nop56	Nop56	BoxC/D snoRNP main component
2	2		Nop58	Nop58	BoxC/D snoRNP main component
2	2	2	FBL	Nop1	BoxC/D snoRNP main component
2	2	11	NHP2L1	Snu13	BoxC/D snoRNP main component
2	2	2	Rrp9	Rrp9	Specific factor of U3 snoRNA
				Mpp10 complex	
8	8	8	MPHOSPH10	Mpp10	Structural
2	2	2	Imp3	Imp3	Same
2	2	8	Imp4	Imp4	«
				Individual factors	
	2	8	DCAF13	Sof1	«
8	8	8	WDR46	Utp7	«
2		2	DNTTIP2	Fcf2	«
2	2	8	FCF1	Utp24	A1, A2 nuclease
1	2		UTP3	Sas10/Utp3	Structural, has the exosome binding motif
2	2	8	UTP11L	Utp11	Structural
				5’-domain	
2	2	8	AATF	Bfr2	Same
2	2	8	NOL10	Enp2	«
2	2	2	NOL6	Utp22	«
				Central domain	
2	8	8	RRP7A	Rrp7	«
8	8	4	PDCD11	Rrp5	«
1	2		Krr1	Krr1	«
1	2		BYSL	Enp1	«
				3’-main domain	
2	2	2	NOP14	Nop14	«
2	2	2	NOC4L	Noc4	«
7	7	7	Rrp12	Rrp12	«
1			NAT10	Kre33	Cytosine acetyltransferase/ helicase
1	2	2	Bms1	Bms1	GTPase
2	2		Rcl1	Rcl1	Structural
1	1		EMG1	Emg1/Nep1	rRNA methyltransferase
4	4	4	RSL1D1	Utp30	Structural
6	6	6	Pno1	Pno1	Same
2	2	8	Utp20	Utp20	«
8	8	4	UTP14A	Utp14	Dhr1 binding
				Rrt14	«
				Faf1	«
				Dhr1	DEAH-box-helicase
2			Nob1	Nob1	D-site nuclease
	5	5	DHX33	Dhr2	DEAH-box helicase
1			DHX35		
1	1		C1orf107	Utp25	Structural
10	10	10	WBSCR22	Bud23	rRNA methyltransferase
			TRMT112	Trm112	Methyltransferase adapter
9	9	9	Ltv1	Ltv1	Structural
		4	Tsr1	Tsr1	Same
		4	RIOK1	Rio1	«
	10		RIOK2	Rio2	«
			CSNK1A1	Hrr25	Casein kinase
	4	8	DIMT1L	Dim1	rRNA demethylase

Approximately 18 out of 60 RAFs in the 90S particle are β-propeller proteins that mediate protein–protein interactions during the formation of macromolecular complexes [110]. In addition, several proteins with Trp and Asp (WD) repeats in 90S bind directly to specific rRNA sites. Another large group of 90S RAFs are α-helical proteins. The large proteins Utp20 (~220 kDa) and Utp10 (~180 kDa) are linked to each other, reaching remote regions on the 90S particle with their long α-helices. For example, Utp10 extends from the base of 90S, where 5’-ETS is located, to the top of 90S (5’-domain), where it binds to the Utp20 wrapped around the head of the 90S particle (Fig. 5, Fig. 6). These distant contacts facilitate communication between different regions and/or promote recognition of a common conformation to coordinate maturation steps [5]. Some 90S biogenesis factors are partially or completely unfolded. These polypeptides are present both on the surface and deep in the 90S subparticles. A typical example is Mpp10, which winds around 90S and comes into contact with Imp3, Imp4, Bms1, Utp12, Utp13 (UTP-B), and some regions of the 18S rRNA (Fig. 5, Fig. 6). Similarly, Nop14 is in contact via its long N- and C-terminal regions with Noc4, Emg1, and Rcl1. These elements not only stabilize the 90S complex, but also participate in long-range interactions and/or in conformational sensing [5].

The last step in the 90S conversion is the detachment of the pre-40S complex. This step is closely related to cleavage of the 35S precursor at the A1 and A2 sites at the first stage of the 60S large subunit precursor biogenesis. Interestingly, Utp24 is in close proximity with the A1 site in the 90S particle but cannot perform its function because another RAF, Sof1, masks the A1 cleavage site. Thus, transition of the 90S pre-ribosome to the next stage of assembly requires significant conformational rearrangements that are a result of interaction between new RAFs (e.g., helicases) and the pre-ribosome and/or hydrolysis of macroergic bonds. In particular, several additional enzymes, such as Kre33 acetyltransferase or Nop1 and Emg1 methyltransferases, are present in the 90S particle. Although RNA helicases are involved in RNA structural rearrangements, including snoRNA dissociation, they are absent in the 90S complex. The 90S to pre-40S transition is stimulated by the helicase Dhr1/Ecm16, because the helicase appears to disrupt the base pairing between snoRNA U3 and pre-rRNA and to be involved in 5’-ETS cleavage [111, 112]. Many factors bind pre-rRNA transiently and only until cleavage at the A2 site. These include small RNAs (U14, snR10, and snR30 [113, 114]) and the proteins associated with each of the 18S rRNA subdomains [115, 116, 117], although their role remains poorly understood (Fig. 5).

The interaction of proteins, such as Mpp10, Utp11, and Sas10 (Fig. 5), and base pairing between the U3 snoRNA and the 5’-ETS and 18S rRNA (Fig. 5) provide additional particle stability, mainly acting as local stabilizers of RNA structural elements [31, 44]. Proteins containing helical repeats (Nop14, Noc4, Rrp5, Utp10, and Utp20) and playing mainly a structural role, as well as some enzymes, such as methyltransferase Emg1 [118], acetyltransferase helicase Kre33 [52], and GTPase Bms1 [31, 52], are located in the outer regions of the SSU processome. The temporal order in which enzymes act on the encapsulated pre-18S rRNA remains to be determined.

**Transition from 90S pre-rRNP to 40S pre-rRNP: Release of 5’-ETS**

Inhibition of the RNA exosome due to a mutation in Utp18 [53] or arrest of 90S assembly on the 3’-truncated pre-rRNA [46, 119, 120] stabilizes the complex of 5’-ETS RNA with UTP-A, UTP-B, U3 snoRNA, and other biogenesis factors, which is released during transition from the 90S to pre-40S subparticle [5, 53]. Degradation of 5’-ETS by the RNA exosome should lead to a recycling of biogenesis factors [90, 91].

Further maturation stages require coordinated cleavage at site A1 of 5’-ETS and A2 of ITS1, which acts as a signal for separation of the 18S rRNA and 5.8S/25S rRNA (Fig. 6) [5, 36, 44].

The dissociation of factors enables the formation of contacts between four 18S rRNA domains, which tightens the structure (Fig. 6). Cryo-EM structures showing the 90S to pre-40S transition revealed seven intermediate pre-ribosomal particles, Pre-A1, Post-A1, Dis-C, Dis-A, and Dis-B, which successively replace each other during biogenesis (Fig. 6E) [121].

In the Pre-A1 state, the helix h21 of the pre-18S rRNA occurs in its matured/correct position (Fig. 6E). Along with cleavage at the A1 site, structural changes result in the formation of the Post-A1 intermediate. Sequential dissociation of several assembly factor modules in the intermediate states Dis-C, Dis-A, and Dis-B leads to gradual simplification of the complex, with the main interactions in the 90S subparticle being preserved. Probably, the decisive step in the disassembly of a 90S intermediate depends on the degree of maturation of the pre-40S domains, which is reflected in its compaction degree. rRNA becomes more compact owing to the remodeling of rRNA and RNP, which enables the formation of the decoding center [44]. The degree of compaction may be a signal for disassembling the 5’-ETS scaffold, as seen from the structures preceding cleavage at A1 [90]. This suggestion is consistent with the dependence of cleavage at A1 on the activity of the helicase Mtr4 that probably remodels 5’-ETS [103]. Turning and displacement of RNA helixes, starting in the 3’-region of 5’-ETS, enable movement of Pno1 and h45 and simultaneous attachment of the helicase Dhr1 that forms part of the rRNA helix h1 required for cleavage at A1 by Utp24 endonuclease. This complex process is accompanied by a dissociation of several factors, further destabilization of the intermediate 90S complex, and displacement of 5’-ETS. This results in release of RNA–protein complexes and the pre-40S formation (Fig. 5) [121].

**Export of pre-40S particles**

Within the 90S complex, the 20S pre-rRNA is formed (Fig. 3). It contains 18S rRNA and part of ITS1. The 20S pre-rRNA is a component of the earliest pre-40S particles. Pre-40S bind to several RAFs (nucleolar protein Tsr1 and cytoplasmic proteins Ltv1, Rio2, and Nob1 (Fig. 5)) and are rapidly transported into the cytoplasm. Due to their large size, pre-ribosomes move through the nuclear pores one at a time. The karyopherin Crm1/Xpo1, with the involvement of Ran/Gsp1, transports them into the cytoplasm in a GTP-dependent manner [122]. Rrp12, together with Crm1, binds to 90S and participates in 35S pre-rRNA processing at the A0 site [123]. A decrease in the level of Rrp12 or Crm1 causes accumulation of the pre-40S complex in the nucleoplasm [124]. At least three RAFs (Dim2, Ltv1, and Rio2) present in pre-40S particles contain predicted or functional nuclear export signals, but none of them alone is necessary for export. The functions of the other factors involved in the export of pre-40S subunits have not been identified.

**Processing of pre-40S subparticles in the cytoplasm**

**Fig. 7:**
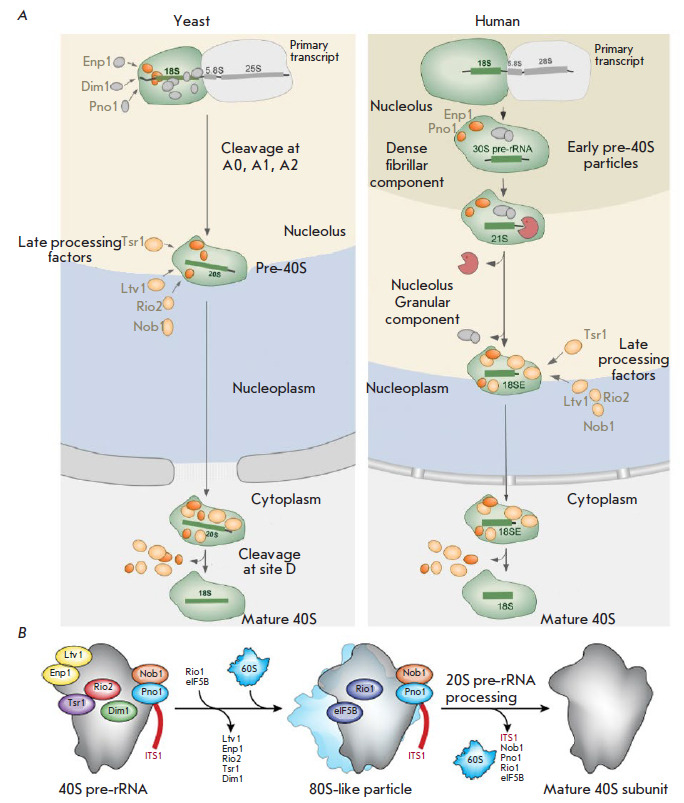
Late maturation stages of the human and yeast ribosomal subunits and subcellular localization of the main assembly participants. (*A*) 40S pre-ribosome intermediates in *S. cerevisiae *(left) and *H. sapiens *(right). Stable identification of two additional pre-rRNAs (30S and 21S) in human cells indicates that there are at least two distinct early maturation stages that are not observed in yeast. Similar compositions of cytoplasmic pre-40S particles suggest similarities in late maturation in yeast and humans. (*B*) Schematic of quality control of the cytoplasmic pre-40S subunit. Only assembly factors with known binding sites are shown [125].

According to biochemical and structural data, pre- 40S particles have a relatively simple RAF composition upon transition to the mature 18S rRNA structure. The first cryo-EM structure of the pre-40S particle revealed almost formed 5’- and central (platform) domains, while the 3’-domain (head and beak regions) had not yet reached a mature conformation. The pre-40S subparticle entering the cytoplasm contains seven RAFs that promote late maturation events (Fig. 7). Two main events occur in the cytoplasm: beak-forming structural rearrangements and 20S pre-rRNA cleavage at the D site by the endonuclease Nob1. They are closely associated with quality control mechanisms and functional site checks, which ensure that ribosomal subunits are translationally competent [125]. Maturation of the beak is facilitated by the release of RAFs and export factors, stable attachment of several ribosomal proteins, and conformational rearrangement that results in the formation of the decoding site. Phosphorylation of the Ltv1 and Enp1 proteins by the kinase Hrr25 allows them to displace and properly place the mature Rps3 protein, which promotes Nob1-dependent 20S pre-rRNA cleavage at the D site [122]

CryoEM data of yeast and human pre-40S particles revealed a significant structural similarity in the positions of associated late RAFs, which occupy functionally important sites and block the formation of functional ribosomes [5, 126, 127, 128]. In particular, RAFs Tsr1, Enp1, Rio2, and Pno1/Dim2 jointly control incompletely formed sites in pre-40S: the decoding center and mRNA-binding groove (Fig. 7). In the early stages, Enp1 and Ltv1 occupy the binding site of ribosomal eS10 in the 3’-major domain (head and beak), dissociating upon phosphorylation by the protein kinase Hrr25 [5, 129, 130, 131]. The dissociation of Enp1/Ltv1 leads to attachment of eS31 and displacement of the C-terminal domain of uS3, which stabilizes the interaction between the 40S body and head [132]. The mechanism of timely cleavage of 20S pre-rRNA by the endonuclease Nob1 may be explained using cryo-EM structures. The RNA-binding protein Pno1 masks a cleavage site at the 3’-end of the mature 18S rRNA. Conformational rearrangement and interaction of the pre-40S subunit with the mature 60S subunit are the checking steps required for interaction with Nob1, which converts the 20S pre-rRNA into the 18S rRNA [5, 38, 133, 134, 135, 136, 137]. A Cryo-EM analysis of human, late pre-40S particles supports a model where Rio1-ATP interacts with the ribosomal protein RPS26 and displaces Dim2 from the 3’-end of the 20S pre-rRNA. This makes the pre-rRNA available for the interaction with Nob1 endonuclease. Hydrolysis of ATP and release of ADP lead to a dissociation of the Rio1–40S subunit complex. The locking mechanism with two keys, Rio1 and RPS26, guarantees consistency in the transformation of particles into translation-competent 40S sub-particles [138]. Coordination of 80S-like particle formation with final maturation of the 18S rRNA ensures that only correctly assembled 40S subunits participate in translation.

Thus, despite the abundance of data for S. cerevisiae and the high conservatism of eukaryotic ribosome biogenesis, the architecture of processing common to both subunits of the 90S precursor and 40S subunit precursor in higher eukaryotes has undergone significant changes, whose details are yet to be studied.

Further description of the large 60S subunit biogenesis will be presented in the next part of the review.
